# Merkel cell carcinoma: long-term outcomes after radiotherapy treatment

**DOI:** 10.1007/s12094-025-04012-x

**Published:** 2025-07-31

**Authors:** Enar Recalde Vizcay, Begoña Navalpotro Yagüe, Carla Ferrandiz-Pulido, Jorge Hernando Cubero, Jaume Capdevila, Blanca Peregrin Pastor, Raquel Granado Carrasco

**Affiliations:** 1 Department of Radiation Oncology, Vall d’Hebron University Hospital, Vall d’Hebron Walk, 119, 08035 Barcelona, Spain; 2 Department of Dermatology, Vall d’Hebron University Hospital, Vall d’Hebron Walk, 119, 08035 Barcelona, Spain; 3 Department of Medical Oncology, Vall d’Hebron University Hospital, Vall d’Hebron Walk, 119, Barcelona, 08035 Spain

**Keywords:** Merkel cell carcinoma, Adjuvant radiotherapy, Radical radiotherapy, Local control, Neuroendocrine carcinoma

## Abstract

**Background:**

Merkel cell carcinoma (MCC) is a rare, aggressive disease with high relapse and fatality rates. Radiotherapy (RT) is essential for local control, but published data on both initial treatment and relapse management remain limited.

**Material and methods:**

We retrospectively analyzed 17 patients treated in a single-center with radical and adjuvant RT.

**Results:**

After a mean follow-up of 44 months, five (29%) patients relapsed (mean time to relapse 5.8 months after RT). Four relapses were locoregional, occurring outside the RT field, and received local salvatge therapy. One relapse was distant. Three patients received up to three consecutive RT courses for locoregional progression without systemic therapy. Five (29%) patients died after a mean time of 35.2 months, four of them with active disease. Two deaths were due to disease progression.

**Conclusions:**

Despite optimal local treatment, relapse remains common and is linked to poor outcomes, such as distant progression and death. Our findings reinforce the need for close postoperative monitoring and early initiation of adjuvant RT. Outcomes after early relapse suggest a potential role for treatment intensification.

## Introduction

Merkel Cell Carcinoma (MCC) is an rare, aggressive cutaneous neuroendocrine carcinoma. It primarily affects elderly white males, and associates with chronic sun exposure and immunosuppression. Up to 80% of the diagnosis are associated with Merkel Cell Polyomavirus infection and most frequent presentation site is head and neck (HN) area, followed by extremities and the trunk [1].

MCC is a dermal proliferation, with epidermotropism found only in 10% of cases. Epidermal stem cells and pre-B cells are thought to be origin of MCC, although it is under debate [2]. Lymphovascular invasion (LVI) and isolated tumor cells far from the main tumor are frequently found, contributing to its aggressiveness with 26% lymph node and 8% distant metastasis rates.

Despite treatment, one-third of patients will relapse, usually within the first two years after disease (80%); and more than one-third may die of disease, surpassing melanoma fatality rate.

Initial staging should include PET-CT, which alters management in nearly half of patients [3]. Sentinel lymph node biopsy (SLNB) is recommended, even in clinically negative patients, due to high rates of occult nodal metastasis, although its use in head and neck tumors is less well established [2].

Surgery is the standard first-line treatment, where traditionally, “wide excision margins” are aimed. This ideally might include a 2 cm margin down to muscle fascia, which is challenging in HN. Insufficient margins increase relapse risk, reported up to 80%. Mohs surgery may be used in certain locations but requires adjuvant radiotherapy (aRT) for optimal control, despite limited evidence [4]. Recent studies suggest narrow excision margins combined with aRT can achieve excellent local control (LC). Narrower excisions are associated with reduced postoperative morbidity and wound complications, which may help minimize delays in initiating aRT. [5].

The role of aRT is well established; some authors support its routine indication as lots of good conditions are needed for considering its omission safe in terms of LC. Optimal timing within eight weeks post-surgery has already being determined [6]. Large retrospective database studies confirm survival benefits from aRT in localized MCC, with no added benefit from adjuvant chemotherapy. However, these studies lack detailed data about type of surgery or time to RT, treated volumes or staging methods [7, 8].

Given MCC’s known radiosensitivity, radical RT (rRT) provides excellent LC with minimal toxicity and is a valuable option for large or anatomically complex lesions, as well as for unfit patients for surgery [9–11]. In addition, relapse after RT can receive successive RT as salvage without expecting significant toxicity [11]. RT can also treat lymph nodes (LN) as an alternative to lymphadenectomy [12].

Since systemic progression often lead to fatal outcomes, improving local management strategies is crucial. However, many retrospective cohorts lack detailed information about previously mentioned factors. Due to MCC’s low incidence, prospective data are limited. Thus, detailed analyses of multidisciplinary management with updated diagnostics and therapies are essential to optimize treatment strategies and improve outcomes.

We present data of MCC patients treated with radiotherapy, providing detailed data on diagnosis, treatment, and follow-up.

## Material and methods

Data about a single-center cohort of 17 patients was retrospectively collected. All patients had biopsy-confirmed MCC and were treated at our institution between 11/2011 and 04/2024 with rRT or aRT. Patients treated with palliative intention were excluded, as well as treatments for late relapses after surgery. Detailed information on all patients is reported, including clinical features, staging investigations, surgical intervention, margins and histology, as well as the time to aRT and the type of radiation received, along with follow-up from diagnosis (biopsy) to death or last clinic visit. How relapses were treated was also registered. Relapse before and after RT were analyzed separately, and classified as loco-regional (LRR; when local or surrounding skin or LN) or metastatic (M). Information on toxicity is available for 13 out of 17 patients and graded according to CTCAE v5 terms [13]. A descriptive analysis was performed, odds ratios (OR) were calculated with MedCalc Software (MedCalc Software Ltd. OR calculator Version 23.1.7) to assess associations. Survival analysis using Kaplan Meier test was made, from first biopsy-confirmed diagnosis to progression, death or last follow-up date. No patient was missed during the follow-up.

## Results

### Diagnosis

Seventeen patients, all caucasian race, aged 55 to 91 years (mean 73.5 years) were included, most (65%) were women (Table 1). Three patients were immunosuppressed (lung transplant, renal transplant and the third one receiving chronic corticosteroid treatment for vasculitis).

**Table 1:** Patient and tumor characteristics at diagnosis

	*n* (17)	% (100)
Age	Mean 73.5 years Median 74 years Range 55–91 years	
Sex		
Male	6	35%
Female	11	65%
Race		
Caucasian	17	100%
Other	0	0%
Immunosuppression		
Yes	3^a^	18%
No	14	82%
Primary tumor location		
Head and neck	10	59%
Extremities	5	29%
Trunk	2	12%
Imaging at diagnosis		
PET-CT	14	82%
CT	3	18%
Primary tumor treatment		
Surgery	13	76%
RT	4	24%

^a^1 lung transplant recipient, 1 kidney transplant recipient, 1 patient with chronic corticosteroid therapy for vasculitis.

Staging PET-scans at diagnosis were performed in 94% of patients. Most (10/17) primary tumors were located in the HN area. None of the patients had distant metastasis at the time of diagnosis. Three patients had clinically positive lymph nodes (two with no known primary). Five had loco-regional satellite lesions.

### Treatment

Thirteen patients underwent surgery as primary treatment. Nine of these interventions were wide excisions, with margins ranging 1–2 cm. Narrower margins correspond to eyelid, scalp, and parotid gland surgeries. The remaining patient had an unknown primary and only underwent lymphadenectomy. Ten patients had negative microscopic margins (R0) and 8 tumors showed LVI. Regarding lymph node surgery, 7/13 underwent SLNB, with 1–4 nodes removed, all negative except from one patient. All except three patients required reconstruction with a flap or graft.

**Fig. 1:**
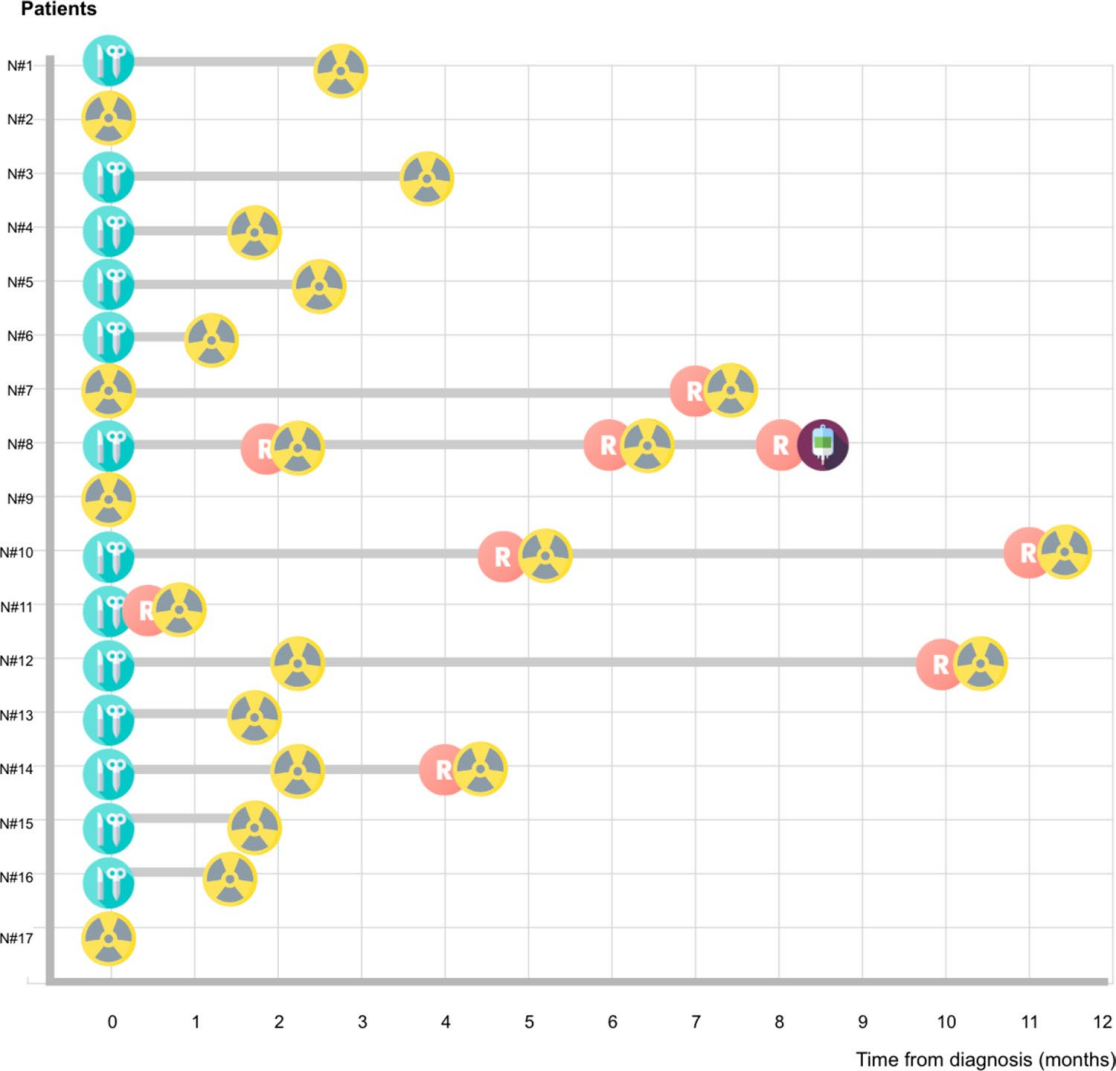
Diagram of initial management for each patient (does not represent total follow-up, only initial management and relapse). Patients numbered from 1 to 15. Time in months from diagnosis. Icons: Blue = Surgery. Red = Relapse. Yellow = Radiotherapy. Purple = Avelumab (colors should be used in printed version). Icons in the Figure 1 designed by Freepik/flaticon, from www.freepik.com and www.flaticon.com

All seventeen patients received photon radiotherapy with VMAT or IMRT techniques (Figure 1). A fixed-thickness soft bolus of 0.3–0.5 cm was used in all treatments. Four patients received it as initial treatment (rRT) and 10 as aRT. The remaining three were patients experienced local recurrence before starting aRT (either awaiting aRT initiation or during postoperative recovery) and were irradiated as both adjuvant and salvage RT (r/aRT). In adjuvant cases, the mean time from surgery to RT initiation was 8.9 weeks and the mean prescribed total dose was 51.4 Gy (range 45–56 Gy). All treatment volumes included the primary tumor or surgical bed (local) when it was known, with 6 including lymph node areas (2/6 elective). Patients with tumor at the time of RT (rRT and r/aRT) were prescribed a higher mean total dose of 59 Gy (range 50–63 Gy) and expansion from GTV to CTV varied from 1.5 to 4.5cm (mean 2.71 cm) (Table 2).

**Table 2:** Data about initial treatment (surgery and/or radiotherapy)

Surgery (*n* = 13 patients)			
Primary tumor resection	Wide	9	69%
	No wide major	4	31%
	Reconstruction with flap	9	69%
Margin status	R1	3	23%
	R0	10	77%
Lymph nodes	SLNB	7	54%
	LDN	3	23%
	LN not surgically explored	3	23%
	pN+	3	23%
	LVI	8	62%
Radiotherapy (*n* = 17)			
RT by intention	Primary radical RT	4	23%
	Adjuvant RT	10	59%
	Radical + adjuvant RT	3	18%
Time from surgery to RT		Mean 8.9 weeks Median 9 weeks Range 3-21 weeks	
RT volume	Local	11	65%
	Local + Ganglionar	5	29%
	Ganglionar	1	6%
Adjuvant RT	Total Dose	Range 45–56 Gy Mean 51.4 Gy	
	Dose per fx	Range 3–2.5 Gy/fx Mean 2.15 Gy/fx	
Radical and Radical + adjuvant RT	Total dose	Range 50–63 Gy Mean 59 Gy	
	Dose per fx	range 1.8–2.5 Gy/fx Mean 2.07 Gy/fx	
	Expansion from GTV to CTV (cm)	Range 1.5–4.5 cm Mean 3.71	
Technique	IMRT	12	71%
	VMAT	5	29%

*SLNB* Sentinel Lymph Node Biopsy, *LDN* Lymphadenectomy, *LN* Lymph Node, *LVI* Lympho-vascular Invasion, *R1* affected surgical margins, microscopic, *R0* free microscopic margins, *pN+* pathologically confirmed positive ganglions, *RT* radiotherapy, Fx fraction, *CTV* Clinical Target Volume, *GTV* Gross Tumor Volume, *IMRT* intensity modulated radiotherapy, *VMAT* volumetric modulated arc therapy

Treatment was well tolerated, with no G4 events being reported. The most frequent toxicity was epithelitis, occurring in all patients, reported as G3 at its worst in 53% of the patients. Three patients experienced local hyperpigmentation and in those requiring cervical irradiation, 3 had G1–2 mucositis. Alopecia happened in scalp locations as expected. Long-term effects included telangiectasias, alopecia, and xerostomia. Two patients presented chronic toxicities requiring surgical resolution: cataracts and eyelid retraction in one same patient, and tympanic membrane perforation in another (Table 3).

**Table 3:** Worse reported toxicity after and during treatment

	*n*	%
Acute toxicity (worse reported grade)		
Epithelitis G1–2	6	35%
Epithelitis G3	9	53%
Hyperpigmentation G1–2	3	18%
Mucositis G1–2	3	18%
Dysphagia G1–2	1	6%
Asthenia G1–2	4	24%
Chronic toxicity		
Telangectasia	3	18%
Xerostomia	3	18%
Disgeusia	1	6%
Alopecia	3	18%
Cataract	1	6%
Tympanic perforation	1	6%
Palpebral retraction	1	6%

Grade according to CTCAE (Common Terminology Criteria for Adverse Events) v5.0

### Follow-up

The mean follow-up time was 44.1 months (range 8–120 months). During this period, 5 patients (30%) experienced disease recurrence (Table 4). The mean time to post-RT progression was 5.8 months (range 2–8 months) from diagnosis. Four out of five patients (80%) presented LRR out of the irradiated field as in transit metastases to the surrounding skin at 2, 7 and 8 months. Only one patient presented LRR to LN (out of field) and only one had visceral progression, both of them had initial nodal involvement and primary tumor located in the lower extremities.

**Table 4:** Detail of patients with relapse (PD)

Patient	Initial site	Initial N+	Surgery	Margins	PD before RT	RT intention	PD site	In/out RT field	Tiime to PD after RT (months)	Treatment of first PD after RT	Further progression after salvage	Death
A	Lower limb	Yes	No	–	–	RAD	LR	out	7	RT	Yes, treated with RT (total RT = 3)	No
B	Lower limb	Yes	Yes	2 cm R0	**yes**	ADJ/RAD	M	Out	6	RT, Avelumab	Yes, treated with systemic therapy	Yes (active MCC, respiratory sepsis)
C	Scalp	No	Yes	2 cm R0	**yes**	ADJ/RAD	LR	Out	6	RT	Yes, treated with RT (total RT = 3)	Yes (active MCC, SARScov2)
D	Lower limb	No	Yes	2 cm R0	no	ADJ	LR	No data	8	RT	Yes, treated with RT (total RT = 3)	Yes (active MCC)
E	Scalp	No	Yes	<1 cm R1	no	ADJ	LR	Out	2	Surgery	Yes, not treated	Yes (active MCC, myeloma)

Margins column: the first number refers to the excision margin width, while the second indicates the microscopic margin status: R0 for negative margins and R1 for positive margins. The fifth death (not represented in this table) was considered cured as relapse never happened after 65 months follow-up

*N+* Nodal involvement, *RAD* radical, *ADJ* adjuvant, *RAD/ADJ* RT after surgery with local relapse, *LR* loco-regional, *M* metastatic, *RT* radiotherapy, *MCC* Merkel Cell Carcinoma

The one with distant metastases had a local recurrence before starting aRT at 4 months, continued with r/aRT, and a PET-scan after completion showed early metastatic progression 2 months later. One patient is considered disease-free at the end of follow-up; the rest died, although only two cases were considered to be due to MCC. Interestingly, two of the patients with relapse after RT had previously relapsed during post-surgery period (prior to aRT).

Following LRR, 3 out of 4 patients received salvage treatment with radical intention radiotherapy (both for the first and second relapses) without having additional systemic treatment or surgical intervention. One of these 3 patients is still alive and disease-free on the last PET evaluation, while the other two died (one from SARS-CoV-2 infection at 34 months and the other at 27 months from MCC). The fourth patient was salvaged with surgery as synchronous progression of myeloma required priority treatment; however, the patient died due to the hematologic disease. The patient with metastatic progression received first line systemic treatment with avelumab and underwent palliative RT to metastatic sites for symptom control and died at 13 months due to progression.

**Fig. 2:**
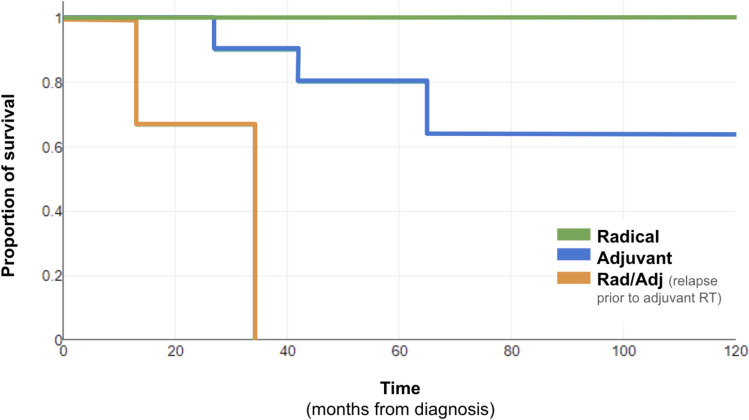
Kaplan–Meier survival curve, death by radiotherapy treatment intention. Time in months after initial diagnosis. Radical (*N* = 4 patients). Adjuvant (*N* = 10 patients). All patients with relapse prior to RT start (*N* = 3) died. None of the patients treated with radical intention RT died (colors should be used in printed version)

After a mean follow-up of 44 months, 5 patients died (Figure 2). The 4-year-OS was 70%. Mean time to death by any cause was 36.2 months (range 65–13 months). One patient was free of disease, the rest had previously relapsed, but only two of them were considered to die due to MCC progression, at 13 and 27 months. One of the 12 patients alive had previously relapsed but 4 of the 5 death patients had a prior relapse of the MCC. The OR of dying was higher if previous relapse (OR = 44, *p* = 0.013). All patients treated with upfront rRT were alive at the end of follow-up.

## Discussion

Our cohort reflects a diverse range of presentations of a rare tumor. A key strength of our study is that nearly all patients underwent PET-CT scans, irrespective of their initial staging or tumor size. PET-CT influenced management in two patients, aligning with the reported potential to alter treatment in up to 40% of cases [3]. Specifically, PET-CT identified a retroauricular tumor in a patient with nodal disease of unknown primary and confirmed N2 extension in a patient with a limb primary. Furthermore, the high rate of surgical nodal assessment in our cohort, coupled with only one patient exhibiting a positive sentinel lymph node biopsy (SLNB) after a negative PET-CT, suggests a high negative predictive value for PET-CT in MCC.

Surgery was the primary treatment for most patients, reflecting common practice, even in challenging areas like the eyelid, where wide margins are difficult to obtain. However, most of the interventions followed the recommendations and achieved at least 1 cm margin excisions, with microscopic margin affection only in 3 patients, yet 2 of these patients suffered early relapse (one before aRT and the other two weeks after aRT), strongly emphasizing the necessity of R0 margins.

Irradiation of uninvolved nodal areas is still controversial. Sometimes can include very wide areas, especially in lower limbs, which creates concern about the possible adverse events. Nevertheless, our data shows very low toxicity in general and it’s important to note that RT-related lymphedema is most often conditioned to post surgery lymphedema, which makes this location maybe also suitable for upfront rRT to cover a wider area and avoid additive toxicity from surgery. Coverage of elective nodal areas seems more “natural” when treating HN locations, as radiation oncologists find it easier and closer to their usual clinical practice as there is much knowledge about lymph drainage derived from HN squamous carcinomas. It is also important to add that SLNB has higher false negative results in head and neck locations, which contributes to support elective irradiation in this location [14].

Head and neck locations were more likely to include elective nodal irradiation (enRT). Unfortunately, a work trying to answer the question about enRT had to be interrupted due to SLNB standardization, and they could not demonstrate significant improvement in survival, although regional relapse probability was significantly reduced [15]. In our work, only one of the 5 relapses was ganglionar and was salvaged with rRT. In another retrospective cohort of 124 patients with localized MCC, enRT happened in 26% of cases but HN cases were less represented (36%) [6].

A large retrospective analysis from Australia reported frequent nodal irradiation (89% of patients, 71% elective) and patients not receiving enRT had much higher rate of nodal relapse (HR = 6.03; 95% CI 1.34–27.10), so they concluded draining nodal areas should be treated in all patients. They interestingly report an 11% rate of nodal recurrence after enRT, much higher than ours [16]. Probably it's because during the study’s period (2000–2005) both PET and SLNB were not standardized, which may explain why we found far fewer recurrences (1/15), even with low enRT treatment and much patients having in transit or nodal metastases at diagnosis.

A prospective comparison of 40 patients with positive nodes receiving lymphadenectomy or radiotherapy reported similar results for both palpable and microscopic node disease. In our cohort 4 patients had initial clinical nodal involvement; two had inguinal lymphadenectomy followed by RT, and the other two (facial primary) had SLNB that resulted negative but received enRT. Only one of the four patients relapsed after RT, which had a palpable inguinal conglomerate at diagnosis, and that conferred worse outcome as shown in Fang’s paper [12].

Primary trunk location is unusual and may lead to late diagnosis with worse outcomes. In our cohort only two patients presented this location but none of them had nodal spread at diagnosis. However, both were routinely explored by dermatologists due to prior multiple skin tumor history (localized SCC, melanoma and others), which probably led to the early diagnosis. This emphasizes the value of specialized follow-up. Nevertheless, those two cases lack sufficient follow-up to draw further conclusions. Additionally, patients with lower limb primary tumors are also rare and in our cohort 3 out of 5 patients experienced relapse when treated for this location.

Out of field “in transit” relapses happening in nearby skin arise concern about the enlargement of treatment volumes and inclusion of elective nodal areas. The current standardized doses, around ≥50 Gy for elective areas and ≥55 Gy for residual lesions seem sufficient looking at the local control rates, as the dose-control correlation found by Foote et al [16]. In our cohort, 3 patients relapsed before aRT, interestingly one was only 3 weeks after a surgery with wide margin excision and has been 21 months free of disease after r/aRT including enRT, but the other two were 9 and 21 weeks post-surgery and presented further relapse after RT. Although the small number of events prevents statistical correlation, they help showing possible natural history of relapsed MCC, where very early relapses can occur before aRT.

Alexander’s work finds significant increasing trend of higher LR with longer time to aRT. In our cohort, mean time to RT was 9.25 weeks, but we observed a reduction in more recent cases (7.25 weeks in cases diagnosed between 2020 and 2023) [6]. In total only 5/12 started RT before 9th week and none of them experienced relapse after RT (OR 14.14 *p* = 0.1). We lack precise data about the reasons for RT delay; it is likely due to multifactorial and may involve the need for better inter-specialty communication, as not so many postoperative complications were reported in our center despite the high number of flap reconstructions.

Multidisciplinary discussion for ideal patient selection is essential, and this may be the reason for the low surgical complications despite the high number of elderly patients with HN wide excisions requiring grafts, and also for the optimal outcomes of the patients who received rRT in our cohort. However, due to the low number of patients treated with only-RT approach, we lack sufficient data to draw conclusions about its effectiveness. In some series recurrences are high (51% out of field, 15% in field) but most included patients who had previously relapsed, which nevertheless shows that rRT is feasible even in the setting of local recurrence and re-irradiation. We managed with rRT 3/5 relapses which received up to 3 rRT to further progressions without needing further systemic therapy [9].

Additionally, reported cases also support the use of rRT for HN relapse, with great cosmetic results as well as excellent progression-free survival (10, 33 and 23 months to each progression). This management is more than feasible, as well as convenient for the patient, as it confers good control with low toxicity when disease progresses with oligo-metastatic pattern, and we’ve seen in our cohort that this is not an unusual presentation of the progressed MCC [17].

## Conclusions

Proper management of MCC requires a multidisciplinary management at a tertiary-level institution with access to PET-CT scans, which enables to decide the best treatment after ruling out hidden metastases, balancing between optimal disease control and least morbidity. Cases suitable for RT-only approach should be identified, particularly when complex surgery or RT delay is expected, as outcomes are good in terms of both toxicity and local control.

Despite following recommendations, relapses are still a challenge. As most occur local and out of field, can be salvaged with both surgery or subsequent RT, but cure is not guaranteed. Nodal relapses are low, even without enRT, probably due to SLNB and PET-CT implementation. Relapses awaiting aRT are not uncommon and seem to indicate a more aggressive disease leading distant spread, reflecting the need vigilant post-treatment surveillance and need for treatment intensification. Even so, factors leading aRT delay must be identified and addressed.

## Data Availability

The data from this study are accessible upon request from the corresponding author.
